# Rib stress fractures in rowers

**DOI:** 10.1186/2052-1847-7-S1-O18

**Published:** 2015-08-11

**Authors:** Anders Vinther

**Affiliations:** 1 Dept. Medicine Herlev University Hospital, Copenhagen, Denmark

## 

Rib stress fracture (RSF) is a relatively frequent, severe and well documented overuse injury in elite rowers. The incidence of RSF has been estimated to be approximately 9 %, but prospective studies with rigorous injury surveillance of larger groups of rowers are needed to confirm this.

The burden of RSFs lies primarily in the severity as most reported cases needed 6-8 weeks of rest, rehabilitation and gradual return to rowing before a full return was possible. Anecdotal reports of non-union of RSF emphasize the necessity of “playing it safe” regarding a timely, symptom dependent return to training and competition.

Since no evidence based therapy is available that can decrease healing times of stress fractures, a RSF can be a season ending injury for an elite rower. Consequently, prevention of RSF is of paramount importance. Effective prevention of sports injuries requires thorough knowledge of incidence and/or prevalence, severity, risk factors and injury mechanisms. Several potential mechanisms of injury have been suggested. The relatively limited data provided in the literature (biomechanics of ergometer rowing) may at best give hints with regards to which of the suggested and rather different mechanisms of injury that are most likely to actually induce detrimental bending forces to the ribs during rowing. The aetiology of RSF has thus been described as multifactorial also referring to the plethora of potential risk factors for stress fractures.

The lack of longitudinal investigations of potential risk factors for RSF in rowing necessitates looking at evidence regarding risk factors for stress fractures in general. In particular, the recent research indicating that low energy availability is of great importance to bone health of endurance athletes seems highly relevant for elite rowers – especially lightweight rowers.

Future prospective studies with continuous surveillance (i.e. internet based questionnaires) of the prevalence of RSF symptoms could elucidate the true burden of RSF. If such an investigation is combined with base-line and intermittent assessment of potential risk factors for RSF stronger evidence for cause and effect relationships could be generated. This type of information could inform effective prevention strategies in the future.

Based on the currently available knowledge from biomechanical studies of ergometer rowing the implementation of dynamic ergometers could be hypothesized to reduce the risk of RSF. Moreover, some of the known risk factors for stress fractures in general might be hypothesized to also contribute to increased bone health and decreased RSF risk in elite rowers.

